# Caffeine Supplementation Enhances Aged Human Oocyte Quality and Embryo Development in In Vitro Fertilization: A Retrospective Paired Study

**DOI:** 10.1002/rmb2.70029

**Published:** 2026-02-13

**Authors:** Jinkyeong Ha, Soyoung Kim, Hyunmee Jang, Yoo Ra Ko, Seyul Han, Woo Sik Lee, Jin Hee Eum

**Affiliations:** ^1^ Department of Obstetrics and Gynecology CHA University Fertility Center Gangnam Seoul Republic of Korea; ^2^ IVF and Fertility Research Lab CHA University Fertility Center Gangnam Seoul Republic of Korea

**Keywords:** caffeine, embryonic development, in vitro fertilization, infertility, maturation‐promoting factor

## Abstract

**Purpose:**

To evaluate whether in vitro caffeine supplementation can improve spindle integrity, embryo development, and clinical outcomes in IVF.

**Methods:**

This retrospective paired‐cohort study included 704 women aged ≥ 35 years who underwent two ICSI cycles (one caffeine‐supplemented and one non‐caffeine) within 12 months. Oocytes in caffeine cycles were exposed to 1.25 mM caffeine for 4 h before fertilization. Outcomes included spindle positioning, embryo quality, blastocyst development and euploidy rates, and neonatal outcomes from 20 women (24 neonates). Paired comparisons were performed using the Wilcoxon signed‐rank and McNemar's tests.

**Results:**

Caffeine cycles showed higher rates of normal spindle positioning (80.2% vs. 61.2%, *p* < 0.001), high‐quality cleavage embryos (63.2% vs. 53.6%, *p* < 0.001), high‐quality blastocysts (39.2% vs. 26.1%, *p* < 0.001), and euploid embryos (22.9% vs. 10.9%, *p* = 0.002) than non‐caffeine cycles. The fertilization rates were similar between the groups (81.4% vs. 80.8%, *p* = 0.628). Clinical pregnancy rate was 39.1%, and neonatal outcomes were reassuring.

**Conclusions:**

Short‐term in vitro caffeine exposure may enhance oocyte and embryo competence in older women undergoing IVF, especially those with prior IVF failures. These findings support caffeine as a simple, practical laboratory intervention. Prospective multicenter studies are warranted to confirm efficacy and safety.

## Graphical abstract

Caffeine supplementation before fertilization is associated with improved spindle integrity, euploidy rates, and embryo quality in oocytes from aged women, with favorable clinical and neonatal outcomes, suggesting a simple and low‐cost strategy that may support IVF outcomes, particularly in older patients or those with repeated IVF failures.

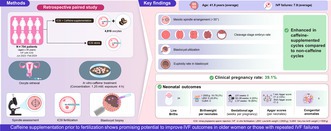

## 1 Background

Infertility is a global health concern, affecting approximately one in six couples worldwide, with advanced maternal age increasingly recognized as a major factor limiting the effectiveness of assisted reproductive technologies, such as in vitro fertilization (IVF) [1]. Age‐related decline in oocyte quality manifests through both structural abnormalities—including aberrant spindle formation and zona pellucida hardening—and molecular dysfunctions such as impaired cell cycle regulation and heightened oxidative stress. Collectively, these alterations diminish fertilization potential and embryo development [2, 3, 4, 5]. The activity of a pivotal molecular regulator of oocyte meiotic maturation, maturation‐promoting factor (MPF), is known to be compromised with oocyte aging [6], and strategies aimed at restoring its activity are therefore of particular interest.

Recent studies have demonstrated that caffeine administration can enhance MPF activity by inhibiting negative regulators such as Wee1 kinase, thereby restoring the active state of MPF in oocytes [7, 8]. In animal models, caffeine supplementation has been shown to improve spindle structure, promote normal pronuclear formation, and increase the proportion of high‐quality blastocysts derived from aged oocytes in various species [9, 10, 11]. Moreover, previous studies using somatic cell nuclear transfer (SCNT) oocytes have demonstrated that caffeine supplementation maintains M‐phase arrest by sustaining MPF activity, thereby improving developmental competence [12, 13, 14].

Although these beneficial effects have been primarily observed in animal models and SCNT oocytes, the applicability of caffeine supplementation to oocytes retrieved for assisted reproductive technology (ART)—particularly in women of advanced maternal age—remains insufficiently explored. Based on these favorable outcomes reported in SCNT models, the present study aimed to investigate whether in vitro caffeine supplementation could confer similar benefits in an ART setting, representing a translational extension of previous findings. Specifically, this study was designed to evaluate whether in vitro caffeine supplementation could (1) ameliorate spindle defects, (2) enhance embryo developmental competence, and (3) improve clinical IVF outcomes. In addition, we assessed the potential effects of caffeine supplementation on neonatal outcomes in women of advanced maternal age with repeated IVF failures, a high‐risk population.

## 2 Materials and Methods

### 2.1 Study Design and Patients

This retrospective observational paired‐cohort study included 704 women who underwent two intracytoplasmic sperm injection (ICSI) cycles at the CHA University Fertility Center Gangnam, between June 2023 and February 2024.

To improve comparability between cycles and minimize potential confounding, only cycles in which ICSI was performed were included. ICSI was indicated according to standardized institutional criteria, including male factor infertility as defined by the World Health Organization (WHO) guidelines, combined male and female infertility factors, previous fertilization failure or low fertilization rates in conventional IVF cycles, or the absence of usable or surplus embryos for cryopreservation.

To rigorously control confounding variables such as aging, environmental exposure, and changes in clinical practice, only patients who completed both a non‐caffeine and caffeine ICSI cycle within a strict 12‐month window were included. In all patients, the caffeine‐treated cycle was performed after the non‐treated cycle. This sequence reflected the stepwise introduction of caffeine supplementation as an adjunctive intervention in routine clinical practice rather than investigator‐driven allocation.

This paired‐cohort design enables direct intra‐individual comparison, thereby controlling for genetic background and baseline ovarian reserve. Cycles conducted more than 1 year apart were excluded to ensure protocol consistency. Eligible patients were aged 35 years or older and had undergone ICSI fertilization. The exclusion criteria were the absence of prior IVF cycles, cycles performed at other institutions, failure or abnormality of oocyte retrieval, abnormal fertilization, or abnormal embryo development by day 2 post‐ICSI.

### 2.2 Ovarian Stimulation Protocol

Ovarian stimulation was primarily performed using gonadotropin‐releasing hormone antagonist and agonist protocols, which were selected based on clinical indications. The medications used in these protocols included a gonadotropin‐releasing hormone antagonist (Cetrotide, Merck, Darmstadt, Germany) and agonist (Lucrin, Dongkuk Pharmaceutical, Seoul, Republic of Korea), and a recombinant follicle‐stimulating hormone (Gonal‐F and Pergoveris, Merck). The dosage of recombinant follicle‐stimulating hormone was adjusted according to each patient's antral follicle count, anti‐Müllerian hormone levels, and previous response to stimulation. The follicular response was monitored via transvaginal ultrasound, and gonadotropin doses were adjusted accordingly. Final oocyte maturation was triggered when the mean diameter of two or more follicles reached ≥ 18 mm, using either recombinant human chorionic gonadotropin (Ovidrel, Merck) alone or in combination with a gonadotropin‐releasing hormone agonist (dual trigger). Oocyte retrieval was performed under conscious sedation at 30–34 h after triggering using ultrasound guidance.

### 2.3 Caffeine Treatment

Immediately after oocyte retrieval, cumulus–oocyte complexes were cultured for a total of 4 h in SAGE Quinn's Advantage Fertilization Medium (CooperSurgical, Trumbull, CT, USA) supplemented with 1.25 mM caffeine (C8960; Sigma‐Aldrich, St. Louis, MO, USA) immediately after ovum pick‐up [12, 13, 14].

The caffeine concentration of 1.25 mM used in this study was selected based on prior evidence from animal studies reporting beneficial effects on MPF activity and spindle stability within the 1–2 mM range, as well as previous experience demonstrating the safe use of 1.25 mM caffeine during human SCNT procedures [9, 10, 11, 12, 13, 14]. Because exploratory dose–response experiments using clinical oocytes from women of advanced maternal age are ethically and practically challenging, this concentration was adopted as a conservative and evidence‐based condition.

At 2 h post‐ovum pick‐up, cumulus cells were removed during the culture period using hyaluronidase (CooperSurgical) and repeated pipetting. To ensure complete removal of caffeine and to standardize the procedure, oocytes were thoroughly washed several times after caffeine exposure prior to ICSI. This washing step was strictly performed to prevent any potential residual effects of caffeine during subsequent embryo culture and development.

### 2.4 ICSI and Spindle Observations

In this study, all cycles were performed using ICSI for fertilization. The procedure was conducted under standard microscopic conditions at 37°C *.* Prior to ICSI, the oocytes were observed for meiotic spindles using PolScope spindle view (Oosight META imaging system; CRI, Woburn, MA) and a glass‐bottom culture dish (MatTek, Ashland, MA). Each oocyte was rotated using an injection pipette until both the meiotic spindle and polar body were clearly in focus on the equatorial plane of the oocyte. The meiotic spindle angle was measured based on the first polar body. Normal spindles were defined as those with an angle < 30°, and abnormal spindles were defined as those with an angle ≥ 30° or those with no visible spindles [15].

### 2.5 Embryo Culture and Grading Procedures

Fertilization was performed using ICSI, and successful fertilization was confirmed by the presence of two pronuclei at 16–17 h post‐ICSI. The resulting zygotes were transferred to SAGE Quinn's Advantage Cleavage Medium (CooperSurgical) and cultured under controlled conditions (5% O_2_ and 6% CO_2_, at 37°C) in Heracell 240i incubators (Thermo Fisher Scientific, Marietta, OH). The oil‐drop culture method was performed using an Oosafe 4 Well Dish (Sparmed, Farum, Denmark).

On day 3, the embryos were assessed for quality and classified as good quality embryos if they contained at least six evenly sized blastomeres, exhibited ≤ 20% anucleated fragments, and showed no evidence of multinucleation. Subsequently, all embryos were transferred to SAGE Quinn's Advantage Blastocyst Medium for further culture. On day 5 and 6, the blastocysts were evaluated using the Gardner grading system, with high‐quality blastocysts defined as those graded AA, AB, BA, or BB [16].

### 2.6 Trophectoderm Biopsy and Genetic Analysis

Blastocyst biopsy was performed on day 5 and 6 at the expanded stage, and embryos with a Gardner score of ≥ 3 BC and well‐defined inner cell mass, blastocoel, and trophectoderm were selected. Blastocysts were placed in biopsy dishes with 10‐μL droplets of 5% HSA/HEPES medium (CooperSurgical) under tissue culture oil (CooperSurgical).

A holding pipette was used to position the blastocyst with the inner cell mass away from the biopsy site. A laser (LYKOS, Hamilton Thorne, Beverly, MA) was used to create an opening in the zona pellucida, and trophectoderm cells were aspirated using a 30‐μm pipette. Cell junctions were cut using a laser, and cells were detached by flicking.

Samples were stored individually per double identification protocol, frozen, and outsourced for ploidy analysis using next‐generation sequencing (CHA Biotech, Seoul, Republic of Korea).

### 2.7 Outcome Measures

Clinical outcomes included rates of implantation, chemical and clinical pregnancy, ongoing pregnancy, live birth, pregnancy loss, and ectopic pregnancy. Clinical pregnancy was defined as the presence of a gestational sac with a fetal heartbeat on ultrasound. Information on neonatal outcomes—including birth weight, gestational age, Apgar scores, mode of delivery, sex, neonatal intensive care unit (NICU) admission, neonatal death, and congenital anomalies—was retrieved from medical records and follow‐up.

### 2.8 Statistical Analysis

Statistical analyses were performed using IBM SPSS Statistics version 29.0 (IBM Corp., Armonk, NY). The normality of data distribution was assessed using the Shapiro–Wilk test. As the continuous variables were non‐normally distributed, comparisons between paired cycles were conducted using the non‐parametric Wilcoxon signed‐rank test. Categorical variables were analyzed using McNemar's test. Results with a *p* < 0.05 were considered statistically significant. Unless otherwise specified, data are presented as mean ± standard error of the mean.

## 3 Results

### 3.1 Clinical Characteristics of the Study Population

A total of 704 patients aged ≥ 35 years were included in this study (Table 1). The mean body mass index was 21.9 ± 0.12 kg/m^2^, and the mean levels of basal follicle‐stimulating hormone and anti‐Müllerian hormone were 9.2 ± 0.21 mIU/mL and 1.3 ± 0.07 ng/mL, respectively. The average duration of infertility was 3.7 ± 0.16 years.

**TABLE 1:** Patient and cycle characteristics.

Characteristic	Value
Number of patients (*n*)	704
BMI^a^ (kg/m^2^)	21.9 ± 0.12
bFSH^b^ level (mIU/mL)	9.2 ± 0.21
AMH^c^ level (ng/mL)	1.3 ± 0.07
Duration of infertility (years)	3.7 ± 0.16
Number of previous embryo transfer cycles	2.4 ± 0.11
Number of previous IVF cycles	7.9 ± 0.21
Cause of infertility (% [*n*])	
Male factor	7.4 (52/704)
Uterine factor	12.4 (87/704)
Unexplained	1.0 (7/704)
Reduced ovarian reserve	2.4 (17/704)
Others	1.4 (10/704)
Combined	75.4 (531/704)

*Note:* in vitro fertilization.

^a^ Body mass index.

^b^ Basal follicle‐stimulating hormone.

^c^ Anti‐Müllerian hormone.

On average, patients had undergone 2.4 ± 0.11 previous embryo transfers and 7.9 ± 0.21 previous IVF cycles. The causes of infertility included male factor (7.4%), uterine factor (12.4%), unexplained infertility (1.0%), diminished ovarian reserve (2.4%), other factors (1.4%), and combined causes (75.4%) (Table 1).

### 3.2 Comparison of Embryonic Development Between the Caffeine and Non‐Caffeine Cycles

The maternal age at the time of oocyte retrieval was 41.8 ± 0.13 years. A total of 4816 and 4950 oocytes were retrieved in the caffeine and non‐caffeine cycles, respectively. The endometrial thickness, number of retrieved oocytes, number of mature oocytes, and fertilization rates did not significantly differ between the cycles. As summarized in Table 2, multiple embryological parameters were significantly improved in the caffeine‐supplemented cycles compared with the non‐caffeine cycles. Specifically, the rates of high‐quality cleavage‐stage embryos (63.2% vs. 53.6%, *p* < 0.001), blastocyst utilization (19.6% vs. 17.2%, *p* = 0.023), and high‐quality blastocyst formation (39.2% vs. 26.1%, *p* < 0.001) were higher in the caffeine cycles than in the previous non‐caffeine cycles (Table 2).

**TABLE 2:** Patient characteristics and embryonic development outcomes between caffeine and non‐caffeine cycles.

	Caffeine cycle	Non‐caffeine cycle (previous cycle)	*p*
Patient characteristic			
Number of patients, *n*	704		
Age (years)	41.8 ± 0.13	41.8 ± 0.13	
Endometrial thickness (mm)	8.9 ± 0.08	8.9 ± 0.08	0.429
Embryonic development			
Number of oocytes retrieved (*n*)	4816	4950	
Average number of oocytes retrieved (mean)	6.8 ± 0.21	7.0 ± 0.22	0.497
Number of MII^a^ oocytes (*n*)	3601	3508	
Average number of MII oocytes (mean)	5.1 ± 0.15	5.0 ± 0.16	0.113
Fertilization rate (%)	81.4 ± 0.79	80.8 ± 0.96	0.628
High‐quality cleavage rate (%)	63.2 ± 1.42	53.6 ± 1.51	< 0.001
Blastocyst utilization rate (%)	19.6 ± 1.09	17.2 ± 0.97	0.023
High‐quality blastocyst rate (%)	39.2 ± 2.46	26.1 ± 2.25	< 0.001

^a^ Mature.

### 3.3 Effect of Caffeine on Meiotic Spindle Localization

**FIGURE 1:**
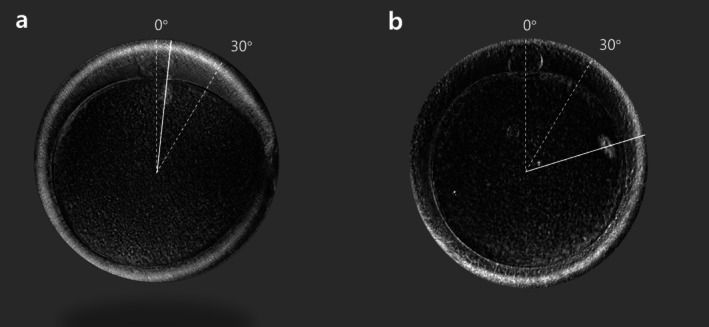
Representative PolScope images of meiotic spindle localization in human oocytes. (a) Normal spindle localization with an angle < 30° relative to the first polar body. (b) Abnormal spindle localization with an angle ≥ 30° relative to the first polar body.

Spindle localization was analyzed in 173 patients, including 735 and 690 mature oocytes from caffeine and non‐caffeine cycles, respectively. The incidence of spindles with an angle < 30° (normal localization) was significantly higher in the caffeine cycle than that in the non‐caffeine cycle (80.2% vs. 61.2%, *p* < 0.001). Conversely, the incidence of abnormally localized spindles (angle ≥ 30°) or absence of visible spindles was significantly lower in the caffeine cycle than that in the non‐caffeine cycle (19.8% vs. 38.8%, *p* < 0.001; Table 3). Representative PolScope images illustrating normal (< 30°) and abnormal (≥ 30°) spindle localization in oocytes from caffeine‐treated and non‐caffeine cycles are shown in Figure 1.

**TABLE 3:** Location and identification of meiotic spindles in caffeine and non‐caffeine cycles.

	Caffeine cycle	Non‐caffeine cycle (previous cycle)	*p*
Number of patients (*n*)	173		
Normal localization^a^ (%)	80.2 ± 2.04	61.2 ± 2.84	< 0.001
Abnormal localization (%)	19.8 ± 2.04	38.8 ± 2.84	< 0.001

^a^ Normal localization: angle < 30°.

### 3.4 Comparison of Euploidy Rates Between Caffeine and Non‐Caffeine Cycles

A total of 97 patients were included in the analysis, with 215 and 242 blastocysts biopsied in the caffeine and non‐caffeine cycles, respectively. The average number of blastocysts biopsied per patient was slightly lower in the caffeine cycle (2.2 ± 0.16) than in the non‐caffeine cycle (2.5 ± 0.15), although the difference was not significant (*p* = 0.063). The euploidy rate (percentage of euploid blastocysts) was significantly higher in the caffeine cycle (22.9%) than in the non‐caffeine cycle (10.9%; *p* = 0.002). The incidence of mosaicism (percentage of blastocysts showing mosaicism) was lower in the caffeine cycle (15.4%) than in the non‐caffeine cycle (21.4%); however, the difference was not significant (*p* = 0.215; Table 4).

**TABLE 4:** Euploidy rates in caffeine and non‐caffeine cycles.

	Caffeine cycle	Non‐caffeine cycle (previous cycle)	*p*
Number of patients (*n*)	97		
Blastocyst biopsy outcomes			
Total blastocysts biopsied (*n*)	215	242	
Average number of blastocysts biopsied (*n*)	2.2 ± 0.15	2.5 ± 0.16	0.063
Euploid (%)	22.9 ± 3.55	10.9 ± 2.10	0.002
Mosaicism (%)	15.4 ± 2.83	21.4 ± 3.23	0.215

### 3.5 Clinical Outcomes After Embryo Transfer in Caffeine Cycles

Clinical outcomes were analyzed for 704 caffeine cycles. Most patients underwent caffeine‐supplemented cycles as repeat attempts following non‐caffeine cycle or total embryo cryopreservation failure; therefore, the number of non‐caffeine cycles available for within‐1‐year paired comparison was limited. Consequently, only the caffeine cycles were analyzed.

**FIGURE 2:**
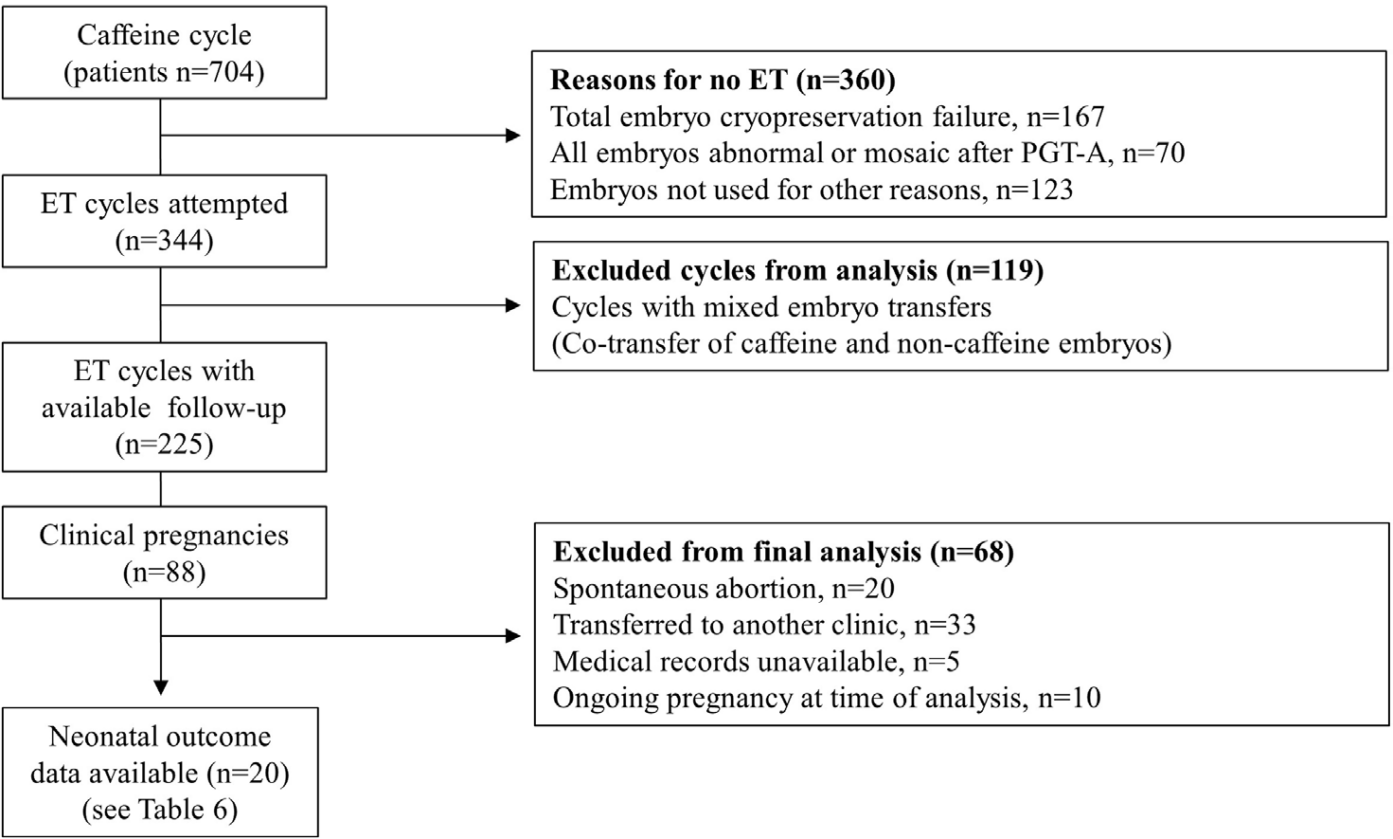
Flow of caffeine‐supplemented in vitro fertilization (IVF) cycles and outcomes. A total of 704 caffeine‐supplemented cycles were included. Embryo transfer (ET) was attempted in 344 cycles. ET was not performed in 360 cycles owing to total embryo cryopreservation failure (*n* = 167), all embryos being abnormal or mosaic after preimplantation genetic testing for aneuploidy (PGT‐A, *n* = 70), or embryos not used for other reasons (*n* = 123). Of the 344 ET cycles, 119 were excluded from analysis because of mixed embryo transfers (co‐transfer of caffeine and non‐caffeine embryos), leaving 225 cycles with follow‐up data. Clinical pregnancies occurred in 88 cycles, and neonatal outcome data were available for 20 women (24 neonates); exclusions included spontaneous abortion (*n* = 20), transfer to another clinic (*n* = 33), unavailable medical records (*n* = 5), and ongoing pregnancies at the time of analysis (*n* = 10).

Of the 704 cycles, 225 were included in the final analysis after excluding cycles for the following reasons: total embryo cryopreservation failure (*n* = 167), all embryos being abnormal or mosaic after preimplantation genetic testing for aneuploidy (PGT‐A) (*n* = 70), embryos not used for other reasons (*n* = 123), and mixed embryo transfers (co‐transfer of caffeine and non‐caffeine embryos, *n* = 119) (Figure 2).

Among the analyzed cycles, the mean number of embryos transferred per cycle was 2.0 ± 0.06. The implantation rate per transferred embryo was 23.0% (103/447), and the chemical pregnancy rate per ET was 4.4% (10/225); clinical pregnancy occurred in 39.1% of ET cycles (88/225). The pregnancy loss rate per clinical pregnancy was 22.7% (20/88), and the ectopic pregnancy rate was 1.1% (1/88) (Table 5).

**TABLE 5:** Clinical outcomes after embryo transfer in caffeine cycles.

Outcome	Value
No. of embryo transfer cycles (ETs), *n*	225
No. of embryos transferred, *n*	447
No. of embryos transferred per ET (mean ± SEM^a^)	2.0 ± 0.06
Implantation rate (% per ET^b^ embryos)	23.0 (103/447)
Chemical pregnancy rate (% per ET)	4.4 (10/225)
Clinical pregnancy rate (% per ET)	39.1 (88/225)
Pregnancy loss rate (% per CP^c^)	22.7 (20/88)
Ectopic pregnancy rate (% per CP)	1.1 (1/88)

^a^ Standard error of the mean.

^b^ Embryo transfer cycle.

^c^ Clinical pregnancy.

### 3.6 Neonatal Outcomes in Caffeine Cycle

Neonatal outcomes were available for 20 women, yielding 24 live‐born neonates due to twin births, after excluding cycles with spontaneous abortion (*n* = 20), transfer to another clinic (*n* = 33), unavailable records (*n* = 5), or ongoing pregnancy at the time of analysis (*n* = 10) (Figure 2). These live births comprised 17 (70.8%) male and 7 (29.2%) female neonates. Birth weight was ≥ 2500 g for 19 neonates (79.2%) and < 2500 g for 5 neonates (20.8%), with no cases of very low birth weight (< 1500 g). Gestational age at delivery was ≥ 37 weeks in 16 pregnancies (80.0%) and 32–36 weeks in 4 pregnancies (20.0%), with no deliveries at ≤ 31 weeks. Twin births occurred in 4 pregnancies (20.0%). The Apgar scores were < 7 at 1 min in 2 neonates (10.0%) and none had an Apgar score < 7 at 5 min, respectively. Cesarean section was performed in 16 pregnancies (80.0%). NICU admission was required for 3 neonates (12.5%); there were no neonatal deaths or birth defects (Table 6).

**TABLE 6:** Neonatal outcomes in caffeine cycles.

Variable	*n* (%)
Number of women analyzed	20
Number of live births	24
Sex (per neonates)	
Male	17 (70.8)
Female	7 (29.2)
Birth weight (per neonate)	
≥ 2500 g	19 (79.2)
LBW^a^ (< 2500 g)	5 (20.8)
VLBW^b^ (< 1500 g)	0
Gestational age, weeks (per pregnancy)	
≤ 31	0
32–36	4 (20.0)
≥ 37	16 (80.0)
Twin birth rate (per pregnancy)	4 (20.0)
Apgar score (per neonate)	
1‐min Apgar score < 7	2 (10.0)
5‐min Apgar score < 7	0
C‐section (per pregnancy)	16 (80.0)
NICU^c^ admission (per neonate)	3 (12.5)
Neonatal death (per neonate)	0
Birth defect (per neonates)	0

*Note:* Data are expressed as *n* (%). Variables are presented per neonate unless otherwise specified (per pregnancy).

^a^ Low birth weight (< 2500 g).

^b^ Very low birth weight (< 1500 g).

^c^ Neonatal intensive care unit.

## 4 Discussion

Caffeine supplementation in the present study was adopted based on its well‐established effects in SCNT models, rather than on direct evidence from human IVF studies [12, 13, 14]. Accordingly, our findings should be interpreted as a translational extension of SCNT‐based concepts to ART settings.

In this study, we demonstrate that caffeine supplementation prior to fertilization improved spindle positioning and embryo development in women of advanced maternal age, with potential implications for clinical outcomes. A retrospective paired analysis of data from 704 patients showed significantly higher rates of high‐quality cleavages and blastocysts, blastocyst utilization, and euploidy, suggesting that caffeine may enhance both the structural and chromosomal integrity of aged oocytes.

Oocyte aging adversely affects the critical structures required for meiosis, including a reduction in the size of the metaphase spindle [17]. Aging disrupts spindle morphology and chromosome movement [18] and interferes with proper microtubule‐kinetochore attachments, increasing aneuploidy rates [19]. Caffeine can restore centrosome integrity and preserve the spindle cytoskeleton in aged oocytes [20]. Consistent with these observations, caffeine‐treated oocytes in the present study exhibited significantly higher rates of normal spindle positioning and euploidy following preimplantation genetic testing for aneuploidy than non‐caffeine‐treated oocytes. These findings indicate that caffeine may mitigate spindle abnormalities associated with oocyte aging, thereby enhancing oocyte and embryo quality.

Mechanistically, these effects are likely mediated through the modulation of the activity of meiotic regulators. In aged oocytes, suppression of MPF activity by Wee1 and myelin transcription factor 1 kinases impairs meiotic progression [21, 22]. Caffeine inhibits these kinases, restoring MPF and mitogen‐activating protein kinase activity, supporting meiotic competence and spindle stability [23, 24]. Additionally, caffeine may enhance intracellular calcium signaling by preserving IP3R sensitivity, maintaining endoplasmic reticulum calcium stores, and supporting mitochondrial function, which are critical to oocyte activation and early embryonic development [25]. Although these aspects were not directly examined in this study, our findings align well with the results of previous animal research. Previous animal studies have demonstrated that caffeine activates meiotic regulators and maintains calcium signaling, thereby preserving the developmental competence of aged oocytes [9, 26]. The improved spindle positioning and increased euploidy rates observed in the present study provide clinical support to the mechanistic insights derived from animal models.

To the best of our knowledge, this is the first study to evaluate the effects of caffeine on aged oocytes in a human clinical setting, advancing prior findings from animal models. Although previous studies in mice, pigs, and cattle have demonstrated that caffeine can restore oocyte quality by enhancing MPF activity and stabilizing the cytoskeleton [9, 10, 11], these findings could not be directly translated to human oocytes because of species‐specific differences in oocyte biology. By bridging the gap between robust preclinical data and clinical application, our findings supply critical, first‐in‐human evidence that supports translation of prior animal research to human IVF practice. The paired study design, with a single patient serving as their own control, further strengthens the reliability of our results by minimizing genetic and inter‐individual variability.

Before this larger‐scale analysis, we also performed a small sibling‐oocyte pilot study (*n* = 30), which showed higher day‐3 high‐quality embryo rates, total blastulation rates, and high‐quality blastocyst rates in caffeine‐treated oocytes (File S1). These preliminary results, retrospectively analyzed as part of routine clinical practice, are consistent with the present study findings and support their reproducibility.

Because both caffeine‐treated and non‐treated cycles were performed in the same patient, direct intra‐individual comparisons were possible while controlling for inter‐individual variability. Additionally, all paired cycles occurred within a 1‐year interval, minimizing potential confounding effects of aging or environmental changes. Our study population comprised patients with poor prognosis, with a mean age of 41.8 years and a history of 7.9 prior IVF failures. In comparison, previous large‐scale studies have consistently shown that advanced maternal age is associated with lower IVF success: in one study of 11,830 cycles, women aged ≥ 40 years had a pregnancy rate of 26.87%, clinical pregnancy rate of 19.39%, and miscarriage rate of 36.14% [27]; another study reported that clinical pregnancy rates decreased sharply with age, with no pregnancies observed in women aged ≥ 45 years [28]. In this milieu, the clinical pregnancy rate per embryo transfer of 39.1% observed in our high‐risk cohort suggests a clinically meaningful improvement in embryo quality and chromosomal integrity, supporting the potential benefit of caffeine supplementation in aged oocytes.

Previous studies investigating caffeine exposure in human oocytes have demonstrated no adverse effects on oocyte morphology or early embryonic development [12, 14]. In our study, neonatal outcomes from 24 live births were generally reassuring, with birth weight, gestational age, Apgar scores, and incidence of congenital anomalies within normal ranges and no neonatal deaths. Although the number of observed live births was small, these preliminary findings, together with previous human data, suggest that short‐term in vitro caffeine supplementation during IVF is unlikely to pose major safety concerns for oocytes or neonates.

Despite these promising findings, certain limitations should be acknowledged. First, this was a single‐center retrospective study, which may introduce selection bias and limit generalizability. Second, the subset of cycles subjected to preimplantation genetic testing and the number of neonatal outcomes were relatively small, restricting the robustness of conclusions regarding euploidy and neonatal safety. Third, although our results are consistent with known mechanisms of MPF activation and calcium signaling, direct molecular assessments in human oocytes were not performed. Finally, long‐term follow‐up of offspring is needed to further evaluate the safety of caffeine supplementation during IVF.

## 5 Conclusions

Caffeine supplementation before fertilization may improve the quality of aged human oocytes and support embryonic development, and appears to be generally safe. Our findings highlight its potential to enhance clinical outcomes, particularly in women of advanced age or those with multiple prior IVF failures. Prospective multicenter trials are warranted to determine optimal protocols and confirm long‐term safety.

## Funding

This work was supported by the Ministry of Science and ICT, Republic of Korea (Project Number: RS‐2023‐00221200; Development of Intractable Disease Therapeutics Based on Stem Cell ATLAS).

## Ethics Statement

This study was approved by the Institutional Review Board of CHA University Fertility Center Gangnam (IRB approval number: 2024‐02‐013).

## Consent

Written informed consent was waived by the Institutional Review Board due to the retrospective nature of the study.

## Conflicts of Interest

The datasets used and/or analyzed during the current study are available from the corresponding author on reasonable request.

## Supporting information


**File S1:** Embryonic development outcomes in sibling oocytes: caffeine vs. non‐caffeine (control) conditions.
Description: Pilot study (*n* = 30) showing day‐3 high‐quality embryo rates, total blastulation rates, and high‐quality blastocyst rates in sibling oocytes with and without caffeine treatment.

## Data Availability

The data that support the findings of this study are available from the corresponding author upon reasonable request.
