# Supplementary material for: Caffeine Supplementation Enhances Aged Human Oocyte Quality and Embryo Development in In Vitro Fertilization: A Retrospective Paired Study
Source: Reprod Med Biol. 2026 Feb 13;25(1):e70029. doi: 10.1002/rmb2.70029 (PMC12903026; doi:10.1002/rmb2.70029)
Supplement: Supplementary file 1 — File S1: Embryonic development outcomes in sibling oocytes: caffeine vs. non‐caffeine (control) conditions. Description: Pilot study (n = 30) showing day‐3 high‐quality embryo rates, total blastulation rates, and high‐quality blastocyst rates in sibling oocytes with and without caffeine treatment. [file RMB2-25-e70029-s001.docx]

**Additional File 1.** Embryonic development outcomes in sibling oocytes: caffeine vs. non-caffeine (control) conditions

|  | **Caffeine** | **Non-caffeine** | ***P*-value** | |
| --- | --- | --- | --- | --- |
| **Characteristic** | |  | |  |
| Number of patients, n | 30 | | |  |
| Age (years) | 37.0 ± 0.71 | | |  |
| AMH^†^ level (ng/mL) | 3.7 ± 0.56 | | |  |
| **Embryonic development** |  |  | |  |
| Number of MII^‡^ oocytes, n | 206 | 202 | |  |
| Average number of MII oocytes, mean | 6.9 ± 0.51 | 6.7 ± 0.63 | |  |
| Fertilization rate (%) | 70.9 ± 4.8 | 73.0 ± 5.0 | | 0.757 |
| High-quality cleavage rate (%) | 36.7 ± 5.8 | 25.2 ± 5.5 | | 0.040 |
| Blastocyst utilization rate (%) | 27.6 ± 4.5 | 26.9 ± 5.5 | | 0.786 |
| High-quality blastocyst rate (%) | 14.5 ± 4.0 | 4.6 ± 2.0 | | 0.034 |

†**AMH** = anti-Müllerian hormone, ‡**MII** = mature
